# The cuter, the better? The impact of cuteness on intention to purchase AI voice assistants: A moderated serial-mediation model

**DOI:** 10.3389/fpsyg.2022.1036848

**Published:** 2022-12-02

**Authors:** Xintao Yu, Zhen Xu, Yifan Song, Xiaochen Liu

**Affiliations:** ^1^School of Economics and Management, Liaoning University of Technology, Jinzhou, Liaoning, China; ^2^School of Communication, East China University of Political Science and Law, Shanghai, China; ^3^Graduate School of Technology Management, Ritsumeikan University, Kyoto, Japan

**Keywords:** AI voice assistant, cuteness, perceived risk, social presence, user engagement, user post-COVID era

## Abstract

Due to the lockdown, more and more people are used to communicating with AI voice assistants during the post-COVID era. This study investigates the relationship between the perceived cuteness of AI voice assistants and the intention to purchase *via* a moderated serial-mediation model. We tested a PLS-SEM model with 284 survey data from an online experiment. The findings indicate that: (1) different cuteness appearances lead to different levels of perceived cuteness; (2) perceived cuteness positively affects intention to purchase; (3) the positive direct effect is serial mediated by social presence and user engagement; (4) the serial mediation effect is negatively moderated by perceived risk of service failure, which means the positive influence of perceived cuteness on intention to purchase is weakened at a high level of perceived risk. Our research has both theoretical and managerial contributions, which also reminds enterprises to grasp the cuteness degree of the product.

## Introduction

Many people now can converse with AI voice assistants on a daily basis, as a result of the rapid development of AI voice-based technologies (Sundar et al., 2017; Li and Sung, 2021). In the United States, sales of AI voice assistants such as Alexa, Cortana, Google Assistant, and Siri reached a record high in 2019 (146.9 million units) and continuing to climb in 2020 (Mishra et al., 2021). Especially during post-COVID era, this kind of human-computer interaction has become more than usual. As people spend more time at home and the use of AI voice assistants becomes more widespread, many psychological and behavioral aspects are affected. AI voice assistant handles over 1 billion daily tasks, most of which are simple information requests or household commands (Dellaert et al., 2020), such as “Cortana, what is the weather today?” “Ok, Google, turn on the lights.” AI voice assistants are changing the lifestyle of consumers, such as seeking service assistance, obtaining information, and purchasing products (Mishra et al., 2021). Romero et al. (2021) considered that AI voice assistants will play an important role in the future.

Previous studies have shown that these intelligent machines are suggested as family members, and the product design of robots determines their acceptance and adoption (Caudwell and Lacey, 2020). Discussion on the product design of robots believes that the baby mode (cuteness) is effective (Tsuburaya et al., 2009; Mara and Appel, 2015). For instance, Lorenz (1971) suggested that the product look of robots, such as large eyes, protruding facial regions, lumbering, stubby limbs, evoke favorable affective responses from humans. This baby-like appearance encourages the establishment of emotional relationships and attachments between human and machine, as well as the desire to hold it in one’s arms and stare at it for extended periods. Shibata (2004) concluded that “cute type animals” and “imaginary cute type animals” performed the most effective function in preventing people’s unfavorable associations. Therefore, cute appearance of product design is usually an important factor to promote the intention to purchase (Lu et al., 2021).

There have been some past studies on the influence of product design of AI voice assistants. Some studies show that the product design of AI voice assistants will affect customer engagement. For example, Muresan and Pohl (2019) explored how users anthropomorphize AI voice assistants and how this influences user engagement. Wang et al. (2020) collected data from 53 college students who feared public speaking and showed that the anthropomorphized sociability of the AI voice assistant increased participants’ satisfaction and willingness to continue engagement. However, previous studies of customer engagement in AI voice assistants rarely considered the impact of cuteness design. Moreover, Chérif and Lemoine (2019) collected data from 640 internet users and found that building trust and social presence in the virtual assistant can generate engagement or adopt intention. Lin et al. (2021) found that social presence could buffer the negative effects of the cute virtual salesperson. However, the relationship between cuteness, social presence and customer engagement is unclear. Lastly, previous research has made few attempts to explore the perceived risk of cute AI voice assistants. Some studies suggest that people worry about the failure of AI voice assistants’ services, especially the cute ones (Lv et al., 2021). But the exact mechanisms of influence are unclear. In general, previous research has not fully captured the cuteness influence mechanism of AI voice assistants.

**Figure 1:**
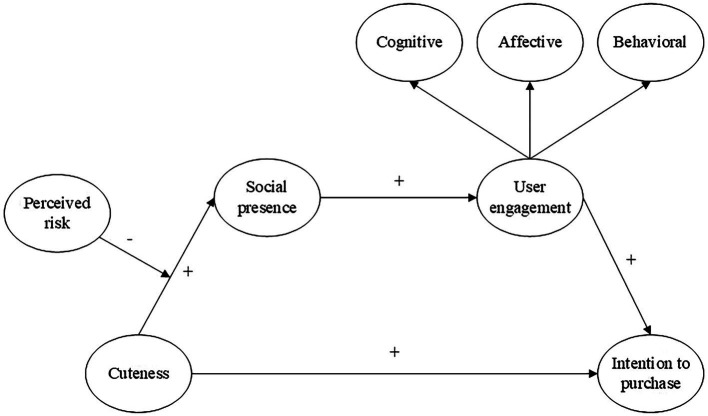
Research model.

To fill the gap, we used an online single factor experiment using different appearance images of AI voice assistants to investigate the influence of consumers’ perceived cuteness on customers’ intention to buy. Moreover, this study examines the chain mediating effect of social presence and user engagement and the moderating effect of perceived risk. We collected 284 survey data by a single factor completely randomized (3 types of cuteness appearances) online experiment and tested a PLS-SEM model. This paper examines the following issues: (1) Does different cuteness appearances design of AI voice assistants can cause different level of perceived cuteness? (2) Does AI voice assistants’ perceived cuteness affect customers’ intention to purchase? (3) Does the social presence and user engagement sequentially mediate the relationship between perceived cuteness and intention to purchase? (4) does perceive risk moderate the sequentially mediating effect? This research can enrich the application of user engagement theory and social presence theory in the AI context. It also helps companies focus on and optimize their AI voice assistants’ cute appearance design and helps AI developers understand human-voice assistant interaction. In Figure 1, we provide the research model.

## Theoretical framework

### Social presence theory

According to Short et al. (1976), social presence is the sensation of being with others. Interpersonal communication led to the evolution of social presence. Social presence is the psychological sensation of being present with perceived intelligence that duplicates the minds of others, given that we can feel present with both real and artificial objects (Tabash et al., 2015). It is vital to keep in mind that social presence is a kind of simulation because it often occurs when the perception machine is another human or non-human intelligence (Biocca, 1997). Biocca et al. (2003) claimed that social presence is not a concept of a physical entity, but a concept of a psychological entity. Another factor that triggers it is the psychologically influenced act of “perceiving presence.” The conceptualization of social presence centers on how individuals perceived their immediate environment. Similarly, Lee (2004) proposed that social presence is a sub-concept of presence, which is a psychological state. In this psychological state, technological users experience the virtual presence of other social actors when they receive certain communication cues.

When a user interacts with a technology, the technological individual can be regarded as a social role of an entity (Nass and Moon, 2000). This is especially true when technological entities mimic attributes possessed by humanoids (Pitardi and Marriott, 2021). Social presence in human-computer interactions has been studied in the past. For example, in the context of e-Products and e-Services, according to Gefen and Straub (2004), social presence has a significant impact on trust and, as a result, online purchase intent. Weisberg et al. (2011) investigated the mediating effect of social presence between past positive experiences and intentions to purchase. Hajli et al. (2017) revealed that familiarity with the platform and the sense of social presence could enhance the propensity to intention to purchase in social networking sites. However, there has not been enough research done on how social presence influences intention to purchase to AI voice assistant.

### User engagement theory

In this study, we choose the definition of (O’Brien and Toms, 2010)’s engagement. Engagement refers to a cognitive, emotional, and behavioral state in which a user interacts with a computer application and wants to stay there. Similarly, Hollebeek et al. (2014) suggested that engagement is a multidimensional concept containing (1) cognitive, (2) affective, and (3) behavioral dimensions. First, the cognitive dimension refers to “a consumer’s level of AI voice assistant-related thought processing and elaboration in a particular human-AI voice assistant interaction.” For example, Foehr and Germelmann (2020) found that users tend to interact with humanoid AI voice assistants. AI voice assistants provide users with the feeling that someone is nearby and can interact with them (Park et al., 2018). Guzman (2019) proposed that users perceived the assistant to be a “distinct entity” based on analyzing smartphone-based voice assistants. Therefore, from the cognitive perspective of the user, AI voice assistant users are more likely to see AI voice assistant as a person who could be perceived as a social presence rather than a device. Second, the emotional aspect refers to “a consumer’s degree of positive AI voice assistant-related effect in a particular human-AI voice assistant interaction.” For example, they will be happy when they treat and interact with an AI voice assistant as a friend (Park et al., 2018), while they will feel angry when the AI voice assistant’s service fails (Lv et al., 2021). Third, behavioral refers to “a consumer’s level of energy, effort and time spent on AI voice assistant in a particular human-AI voice assistant interaction.” For example, Lopatovska et al. (2019) found that when users are bored, they interact with an AI voice assistant, asking it to tell a joke or play a game.

Although “engagement” has received a lot of attention in several disciplines, such as social psychology, the concept has only recently emerged in marketing research (Brodie et al., 2011; Leeflang, 2011). In this field, “engagement” is regarded as a very promising construct, which is expected to provide stronger predictive and explanatory power for focusing on consumer behavior (Schau et al., 2009; Hollebeek et al., 2014). Such as, Shin and Mattila (2021) showed that users will trigger prosocial responses when viewing cute pictures and listening to cute sounds. They suggest that cuteness spur consumer engagement in prosocial practice, increasing consumers’ favorable responses toward a company. Lee and Hsieh (2019), through investigating mobile instant messaging Apps and found that cuteness can project a favorable social image to consumers that facilitate brand engagement, which leads to their willingness to purchase the brand. Jin et al. (2017) found that the higher the degree of user engagement, the closer the relationship between users and others, and thus the more willing to make contributions to others. Therefore, it is necessary to explore the mediation mechanism of user engagement in the AI voice assistant environment.

## Hypothesis

### Cuteness product appearance

Cuteness is described as a special type of attractiveness, which is a subjective perception concept influenced by the visual features of a product’s design (Nenkov and Scott, 2014), such as cuteness in appearance and sound. Academically, Lorenz (1943) made the first study on cuteness within psychology, coming up with the concept of the baby schema. This concept generalizes visually perceived features of a baby’s appearance or behavior, such as, big head, big eyes, small nose, and small mouth. It became the basic design feature of the later cuteness appearance. Subsequently, cuteness has also gained wide attention in various disciplines. For example, In the psychology area, Sherman and Haidt (2011) suggested that cuteness may set up social engagement. In the cognitive science area, Borgi and Cirulli (2016) further found that that juvenile eyes or animal shapes trigger consumer’s perception of cuteness, then stimulating intention to purchase.

In current research on perceived cuteness through product appearance, Borgi and Cirulli (2016) suggest that cute faces can quickly and unconsciously capture the attention of others, thereby triggering positive behavior in people. Lu et al. (2021) showed that cute products attract consumers and it is a positive consumer perception. Nenkov and Scott (2014) found that cuteness is a characteristic based on appearance. However, the mechanism of perceived cuteness for different AI voice assistants’ appearances is unclear. Therefore, we assume:

*H1*: Different AI voice assistant appearances have different influence on consumers’ perception of cuteness.

### Cuteness and intention to purchase

For A long time, researches on the appearance influence of human-computer interaction have been influenced by the schema proposed by Konrad Lorenz (Caudwell and Lacey, 2020). For example, Song et al. (2021) studied that baby schema features of social robots can influence consumers’ trustworthiness perception. The results indicated that people are inclined to choose robots with baby schema features. Mara and Appel (2015) found that higher scores for attributed cuteness when the robots conveyed a head tilt will lead to a high evaluation of robots. Yu (2020) found that people are more likely to accept robots with animated features.

There has been found that the appearance of a product influences consumer product choice (Creusen and Schoormans, 2005). Based on consumer perception, Lu et al. (2021) showed that cute appearance leads to empathy and increased brand trust, increasing intention to purchase. Chou et al. (2022) found that incorporating elements of cuteness into product design resulted in stronger purchase intentions and improved consumer attitudes toward the product. In AI voice assistant research, McLean and Osei-Frimpong (2019) suggested that designers consider providing a look that matches the user’s home design. Similarly, Caudwell and Lacey (2020) believed that cuteness plays an important role in the sales related research of AI voice assistant. However, there is still a lack of AI voice assistant research on whether the cute appearance of AI voice assistants affects purchase intentions. Thus, we hypothesized that:

*H2*: Perceived cuteness positively affects intention to purchase.

### Mediating effect: Social presence and user engagement

Cute facial features have been classified as a highly biologically relevant category of stimuli that can quickly and unconsciously capture consumers’ attention and trigger affectionate responses (Borgi and Cirulli, 2016). Nenkov and Scott (2014) suggested that product attributes can activate subsequent engagement behaviors like individuals, social groups, symbols, and brands. Cute pictures and sounds trigger prosocial responses in consumers, which increase their intention to purchase (Shin and Mattila, 2021). Cuteness spur user engagement in prosocial practice, increasing consumers’ favorable responses toward a company. Lee and Hsieh (2019) investigated mobile instant messaging Apps and found that cuteness can project a favorable social image to consumers that facilitate brand engagement, which leads to their willingness to purchase the brand. Similarly, Jin et al. (2017) showed that the intention to purchase on virtual products is influenced by user engagement. Thus, we hypothesized:

*H3*: User engagement mediates the relationship between perceived cuteness and intention to purchase.

Perceived presence, such as mutual comprehension and minimal standards of decency, is the source of social presence (McLean et al., 2021). These traits have been discovered to promote favorable feelings and social bonds with clients. Such as, Kringelbach et al. (2016) has found that cuteness may facilitate sociality. According to social presence theory (the feeling of being with other people), the cuter the product’s appearance, the more consumers can feel its existence. The fragile character of cute objects might evoke consumer pity, would influence feelings of parental care and affect the adoption intention (Volk and Quinsey, 2002). Similarly, Aradhye et al. (2015) found that smiling children were rated cuter and more likely to be adopted. Therefore, we speculated that the higher the cuteness has, the stronger the presence of the product is, and the easier it is to be adopted by consumers.

Furthermore, Pongpaew et al. (2017) found that high social presence functions affect customer engagement on cognitive, emotional, and behavioral levels. Social presence can directly or indirectly influence consumers’ experience of using high-end technology products (Wagner et al., 2019). Sherman and Haidt (2011) hold that cuteness may set up social engagement, to explore the mechanism by which users interact with cute voice assistants. Therefore, the cuteness of product attributes also affects user engagement to a certain extent, such as AI voice assistant is a product based on human-machine interaction. Consumers can feel it as a friend by talking with it. Thus, we propose:

*H4*: Social presence mediates the relationship between perceived cuteness and user engagement.

User engagement between social presence and intention to purchase to AI voice assistants have proved in previous studies. Such as, McLean et al. (2021) confirmed the importance of social presence influencing consumer brand engagement and validated the role of this construct in AI voice assistant purchase intention. Besides, Kringelbach et al. (2016) found that perceived cuteness positively influences social presence. However, it remains unclear whether the impact of perceived cuteness on intention to purchase can be explained by social presence and user engagement. Therefore, we expected that social presence and user engagement are potentially important and as sequential mediators that can explain the relationship between cuteness and intention to purchase. Specifically, the cuter the AI voice assistant’s product appearance has, the more likely users are to increase their willingness to interact, thus enhancing their perception of the AI voice assistant’s social presence. In turn, the higher level of user engagement with AI voice assistant will then increase consumers’ intention to purchase of AI voice assistant. Therefore, we proposed:

*H5*: The relationship between perceived cuteness and intention to purchase is sequentially mediated by the social presence and user engagement.

### Moderating effect of perceived risk

Perceived risk refers to consumers’ perception of the uncertainty and adverse consequences of the products or services purchased (Dowling and Staelin, 1994). There are many types of such constructs, such as performance, financial, temporal, psychological, and social risk (Lai-Ming Tam, 2012). Perceived risk in this study refers to the performance risk perceived by consumers when using AI voice assistants. Performance risk is defined as the psychological perception of the possibility of disappointment when a product does not live up to consumers’ expectations (Lee and Moon, 2015).

Performance risk in studies related to technology becomes crucial (Trivedi, 2019), such as tolerance of service failure to AI voice assistants. Trivedi (2019) showed that as AI voice assistant is a new technology, the user may have performance risk perceptions, which may have an impact on the user’s feeling about using the technology, leading to a decrease in users’ interaction when perceived risk is high, which means social presence is low when perceived risk is high. In additional, Lv et al. (2021) proposed the tolerance of service failure is positively affected by cuteness, which means that the more cuter products, the more they can mitigate the dissatisfaction of failure of service, which maintain the interaction. However, although cute products can bring people a higher tolerance for service failure, they will also be accompanied by doubts about their function (Li et al., 2021), which means cuteness is antithetical to perceived risk. Attention is a limited resource for everyone (Xia et al., 2020; Ouyang et al., 2022), consumers will be less likely to perceive cuteness if they allocate too much energy to perceive the risk of service failure, where it led social presence lower. Thus, we can speculate that when the perceived risk of service failure is high, the cuter the product is, the service failure will weaken the positive impact on social presence, and consumers will be less likely to interact, thus, affecting intention to purchase, and vice versa. Thus, we hypothesized:

*H6*: Perceived risk moderates the influence of perceived cuteness on intention to purchase through the social presence and user engagement in serial; the sequential indirect relationship will be lower for higher perceived risk.

## Methodology

### Experimental design

**Figure 2:**
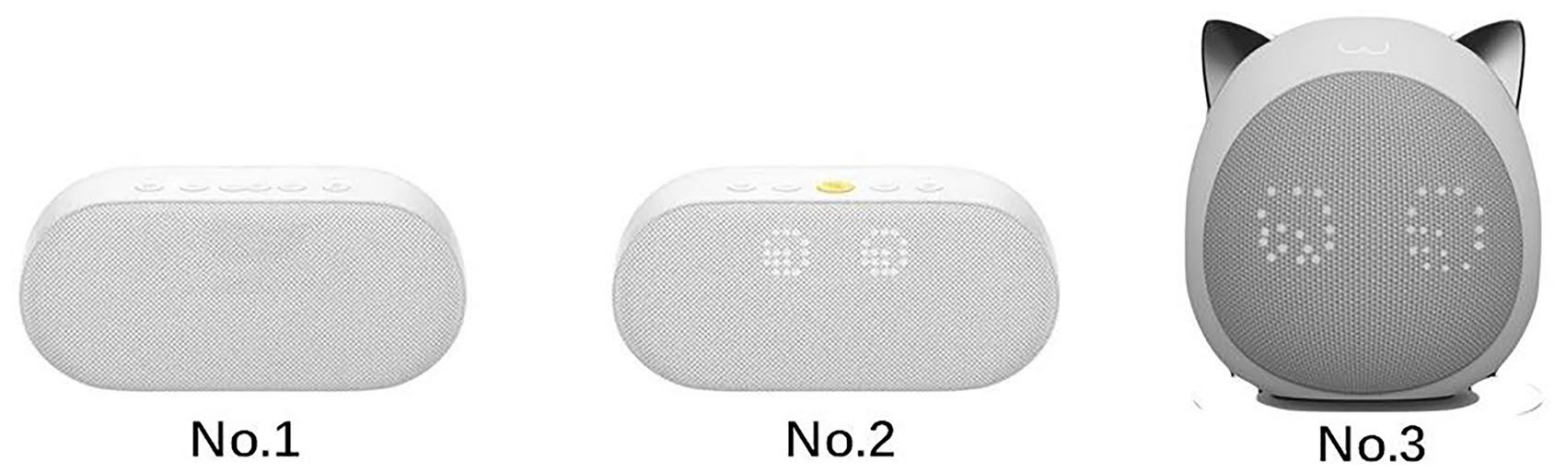
Experimental material.

A single factor completely randomized (3 types of cuteness) experiment design was created for examination to verify the research model. The first AI voice assistant (No. 1) looks like the most common speaker. The second AI voice assistant (No. 2) has a pair of cute round eyes on the speaker. The third AI voice assistant (No. 3) looks like a cute cat, which has a pair of ears, see Figure 2. China’s market for artificial intelligence voice assistants has expanded rapidly in recent years, with a mushrooming of related products. Consequently, Chinese users constitute the study sample. With an experimental approach, questionnaires were distributed to interviewees with experience using AI voice assistants to test the research model. This study conducted an experimental survey regarding the AI voice assistants (named Xiaoxin) that participants were considering purchasing. To improve the efficacy of the experiment, we refer to Lu et al. (2021)‘s research methodology, and the questionnaire was pre-tested before conducting the main survey. The experimental design includes three phases: First, the designed questionnaire system will divide participants into two groups automatically and at random. Then, the system will inquire whether participants have experience using AI voice assistants. If not, the system will remove it automatically. Second, the participants were directed to a website including a questionnaire, where they were required to see video advertisements regarding AI voice assistants. Third, participants began completing the questionnaire based on their feelings after viewing the advertisements. In the video, the tester will first look at the product picture of “Xiaoxin” while hearing a boy say, “Xiaoxin, I am back.” Then hearing Xiaoxin say, “master, welcome home, will open home mode for you. Today is your birthday, master. Xioaxin wishes my master happy birthday!” and then Xiaoxin will play the happy birthday song while starting the smart devices at home (such as turning on the lights in the living room and turning on the TV).

### Participants

The questionnaire was completed using Credamo (www.credamo.com), which is a famous questionnaire website in China. The data collected by us are all approved by the participants, and are only used for research and analysis, without personal privacy and moral and ethical issues. We collected 306 consumers from China who use AI voice assistants. Participants were offered a small financial incentive to participate in the survey. After deleting those answers that contained missing values, the final data contained 284 answers. Table 1 provided an overview of the sample data. In this study, 61.6% of participants were male, 38.4% of participants were female. 3.5% were 20 years old and under, 60.9% were 21–30 years old, 21.5% were 31–40 years old, 8.8% were 41–50 years old, 5.3% were 51 years old and over. 10.6% were high school and under, 62.7% were undergraduate, 23.6% were Master’s degree, 3.2% were Ph.D. degree. The monthly income between 1,000 RMB and under was 7.0%, 1,001–2000 RMB was 22.5%, 2001–3,000 RMB was 15.8%, 3,001–5,000 RMB was 20.8%, 5,000 RMB and over was 33.8%.

**Table 1:** Demographic data.

Profile	Items	Frequency	Ratio (%)
Gender	Male	109	38.4
	Female	175	61.6
Age	20 and under	10	3.5
	21–30	173	60.9
	31–40	61	21.5
	41–50	25	8.8
	51 and over	15	5.3
Education	High school and under	30	10.6
	College	178	62.7
	Master’s	67	23.6
	Doctorate	9	3.2
Disposable income/month (RMB)	1,000 and under	20	7.0
	1,001–2,000	64	22.5
	2,001–3,000	45	15.8
	3,001–5,000	59	20.8
	5,000 and over	96	33.8

### Measurement

The 23 items from previous studies were used to quantify each of the constructs in Table 2. We used a five-point Likert scale [strongly disagree (1) to strongly agree (5)] to evaluate each construct. The researchers enlisted the help of numerous professors in consumer behavior to validate the correctness of the items’ phrasing and substance. The first author, as a multilingual Chinese researcher, translated the scale into Chinese using a translation/back-translation method (Jones et al., 2001). after which multiple language research assistants retranslated the material (Chinese) to ensure the correctness of the translation. To achieve higher reliability and validity of the experiment, we used the scale created in the previous study and modified it properly according to our research demands. We chose Nenkov and Scott (2014)‘s Cuteness © scale and changed it to some amount as the independent variable. The dependent variable of intention to purchase (IP) was adopted from Hsiao and Chen (2018). The mediating variables are adopted from Mishra et al. (2021)‘s social presence (SP), Hollebeek et al. (2014), and Pal et al. (2021)‘s user engagement (UE), which included three sub-constructs (Cognitive, Affective, and Behavioral dimension). We use the repeated indicator approach to deal with the second-order construct of user engagement (Wilson and Henseler, 2007). In other words, all of the measurement items of the first-order construct are used for both the first-order and second-order constructs. Refers to the study of Helme-Guizon and Magnoni (2019), user engagement is a second-order reflective construct designed by three first-order latent constructs including cognitive, affective, and behavioral engagement. Finally, the moderating variable of Perceived risk (PR) takes the scale of Trivedi (2019).

**Table 2:** Results of the measurement model.

Construct	Outer loadings	Cronbach’s *α*	C.R.	AVE.
Cuteness (Nenkov and Scott, 2014)	0.937	0.929	0.955	0.876
	0.943			
	0.928			
Social presence (Mishra et al., 2021)	0.935	0.932	0.956	0.880
	0.951			
	0.928			
Perceived risk (Trivedi, 2019)	0.805	0.812	0.887	0.725
	0.891			
	0.855			
User cognitive engagement (UCE) (Pal et al., 2021)	0.882	0.921	0.944	0.809
	0.909			
	0.911			
	0.895			
User affective engagement (UAE) (Pal et al., 2021)	0.900	0.915	0.940	0.797
	0.894			
	0.896			
	0.881			
User behavioral engagement (UBE) (Hollebeek et al., 2014)	0.931	0.931	0.956	0.878
	0.951			
	0.939			
Intention to purchase (Hsiao and Chen, 2018)	0.930	0.916	0.947	0.856
	0.905			
	0.941			

## Results

We investigated the data set, using ANOVA and PLS. We employed SPSS 27 to examine the impact of 3 different products’ appearance of “Xiaoxin” on perceived cuteness. The research model was conducted using the Smart-PLS. First, the PLS supports exploratory research initially (Gefen et al., 2000). Second, the PLS is an excellent choice because the user engagement is a second-order construct in this study (Hair et al., 2019). Lastly, the PLS can predict with smaller sample sizes (Hair et al., 2011).

### ANOVA analysis

**Figure 3:**
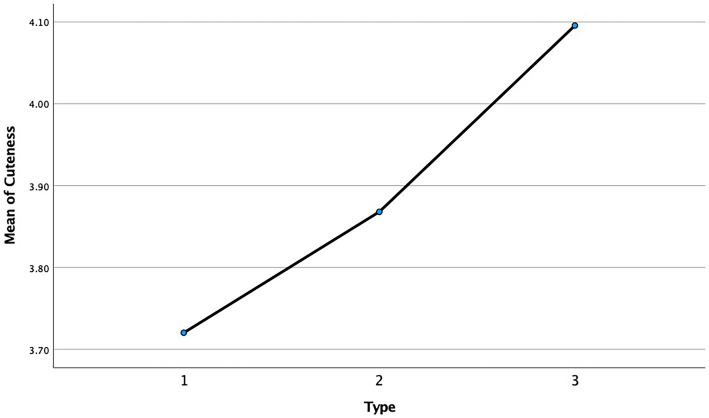
Mean plot.

We first conducted one-way ANOVA (“Xiaoxin” of three different product appearances) with SPSS to evaluate the perceived cuteness of “Xiaoxin.” The result found significant discrepancies between the 3 types of products (*F* = 6.047, *p* < 0.01, see Table 3). Therefore, H1 is accepted because of these findings. In addition, Figure 3 generated by SPSS 27, showed that participants have the lowest perception cuteness of No. 1 and the highest perception cuteness of No. 3.

**Table 3:** ANOVA results (perceived cuteness).

	Sum of squares	df	Mean square	*F*	Sig.
Between groups	6.752	2	3.376	6.047	0.003
Within groups	156.883	281	0.558		
Total	163.635	283			

### Assessment of the measurement model

For the reliability testing, Cronbach’s Alpha (0.70), composite reliabilities (0.80) and average variance extracted (AVE) (0.50) were, respectively, used to evaluate the adequacy of the measurement model, as shown in Table 2. The results show that these measures are effective (Fornell and Larcker, 1981).

For the validity testing, discriminant validity was conducted. First, the square root of the AVE of each construct is greater than the correlation between that construct and other constructs (Fornell and Larcker, 1981). However, in the case of second-order constructs, it is a foregone conclusion that correlations between second-order structures exceed the square root of AVE because second-order constructs use first-order indices (Hair et al., 2019), see Table 4. Furthermore, we also checked H.T.M.T, which showed that each construct does not exceed 0.85, which means that the model has good discriminant validity. See Table 5. Therefore, the model has good reliability and validity.

**Table 4:** Discriminant validity.

	Mean	SD	*C*	SP	UCE	UAE	UBE	IP	PR
Cuteness	3.91	0.76	**0.936**						
Social presence	3.45	0.94	0.572	**0.938**					
User cognitive engagement	3.75	0.77	0.648	0.688	**0.899**				
User affective engagement	3.69	0.75	0.637	0.742	0.766	**0.893**			
User behavioral engagement	3.55	0.81	0.551	0.662	0.747	0.775	**0.937**		
Intention to Purchase	3.39	0.87	0.566	0.637	0.710	0.737	0.730	**0.925**	
Perceived risk	3.34	0.87	−0.128	−0.206	−0.043	−0.086	−0.087	−0.197	**0.851**

**Table 5:** Discriminant validity (H.T.M.T.).

	Mean	SD	*C*	SP	UCE	UAE	UBE	IP	PR
Cuteness	3.91	0.76							
Social presence	3.45	0.94	0.614						
User cognitive engagement	3.75	0.77	0.699	0.813					
User affective engagement	3.69	0.75	0.689	0.807	0.834				
User behavioral engagement	3.55	0.81	0.592	0.711	0.807	0.839			
Intention to purchase	3.39	0.87	0.610	0.801	0.771	0.801	0.788		
Perceived risk	3.34	0.87	0.141	0.098	0.066	0.098	0.098	0.228	

### Assessment of the structural model

**Figure 4:**
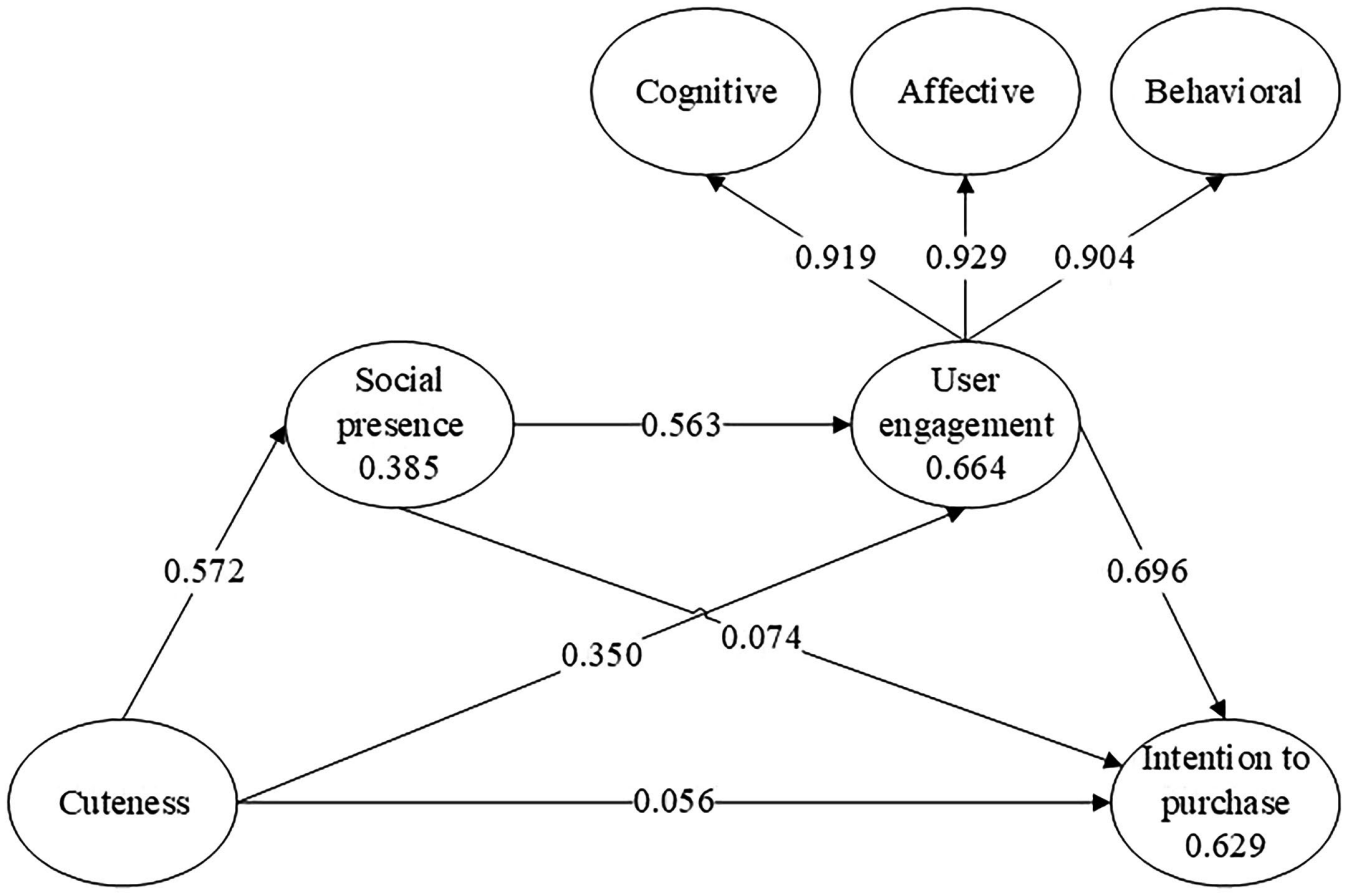
Path coefficient.

Based on evaluating the measurement model, we continue to discuss the structural model. First, To ensure that the model is interpretable, R2 must be as large as possible (Newsted et al., 1998). As shown in Figure 4, the R2 values of social presence (0.385), user engagement (0.664), and intention to purchase (0.629) showed the fit of the overall model is good.

Using the Stone-Geisser Q2 and blindfolding methods, we then assessed the model’s ability to accurately forecast the future. The Stone-Geisser Q2 values of 0.284 > 0 (social presence), 0.455 > 0 (user engagement), and 0.528 > 0 (intention to purchase), demonstrated an acceptable level of predictive relevance (Hair et al., 2016).

Table 6 demonstrates the output results. To validate all of the model’s hypotheses, we resampled the data 5,000 times using bootstrapping. As presented in Table 6, the total effect of perceived cuteness on intention to purchase is significant and positive (*β* = 0.558, *p* < 0.001). Thus, H2 was supported.

**Table 6:** Summary results for the moderated mediation model.

Variable	Social presence	User engagement	Intention to purchase
Cuteness	0.555 (0.557)***	0.350 (0.066)***	0.556 (0.049) 0.521
Social presence		0.563 (0.059)***	0.074 (0.057) 0.194
User engagement			0.696 (0.063)***
Perceived risk	−0.103 (0.054)***		
Perceived risk*Cuteness	−0.249 (0.083)**		
*R*-square	0.385	0.664	0.629
Indirect effect 1	Cuteness → User engagement → Intention to purchase = 0.243; [0.155,0.366]		
Indirect effect 2	Cuteness → Social presence →User engagement = 0.312; [0.235,0.390]		
Indirect effect 3	Cuteness → Social presence → User engagement → Intention to purchase = 0.218; [0.150,0.295]		
Total effect	Cuteness → Intention to purchase = 0.558 (0.056) ***		

*p*-value: *** < 0.001; ** < 0.01.

### Assessment of the serial-mediation hypotheses

We further found that cuteness related to intention to purchase through two significant mediating paths: cuteness → user engagement → intention to purchase; 0.243, [0.155, 0.366], and cuteness→ social presence → user engagement; 0.312; [0.235, 0.390]. Thus, H3 and H4 were supported (see Table 6). Meanwhile, we found that user engagement mediated between social presence and intention to purchase (0.392, [0.272, 0.516]). The result showed a serial-mediation path (cuteness → social presence → user engagement → intention to purchase 0.218, [0.150, 0.295]). Thus, H5 was supported.

### Assessment of the moderator hypotheses

**Figure 5:**
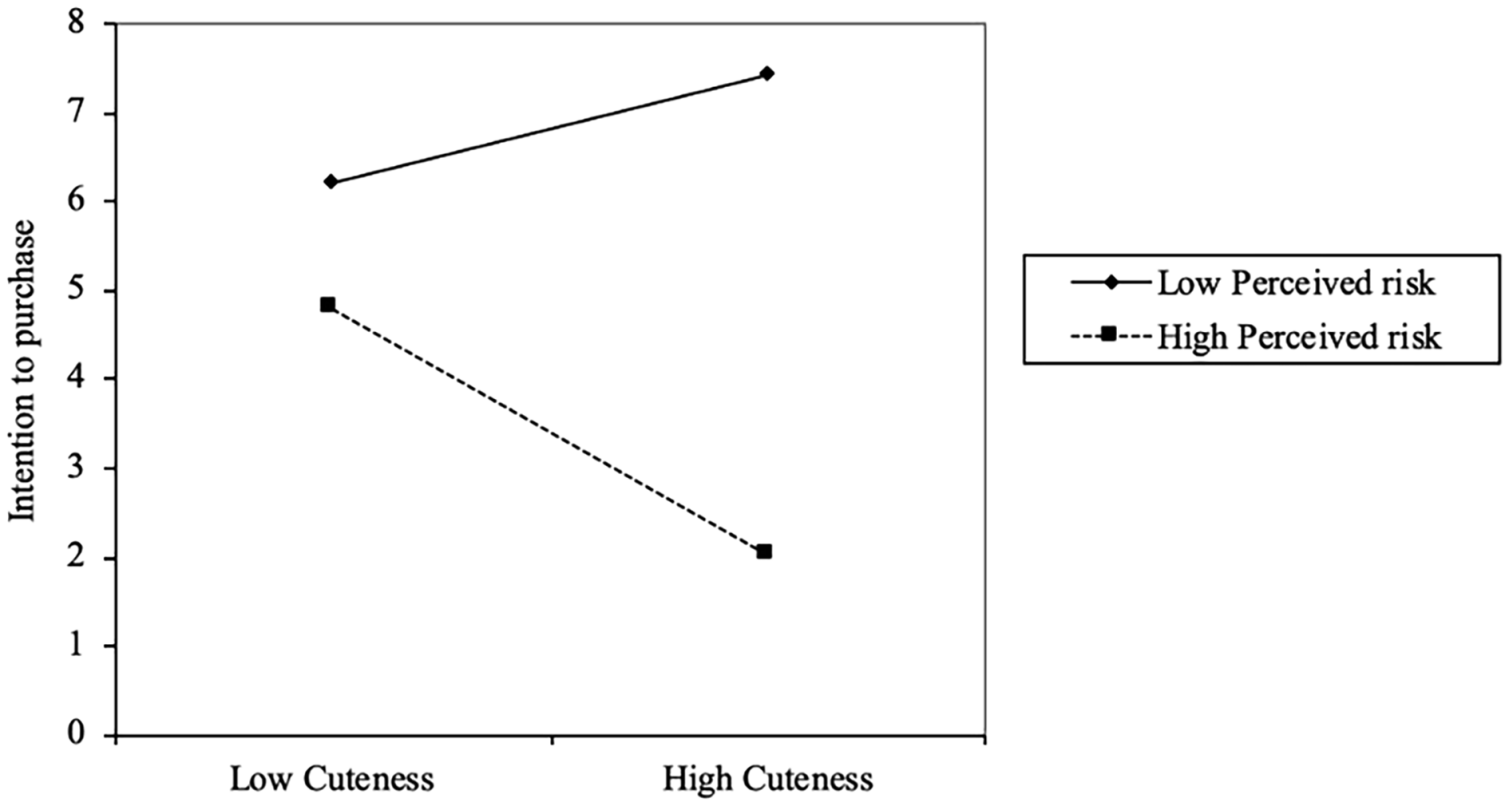
Moderating effect (Perceived risk).

The moderating effect (perceived risk) on the serial mediation model is statistically significant and negative (−0.249, 95% B.C.C.I. [−0.400; −0.080]; see Table 6). As shown in Figure 5, when the perceived risk is high, the positive effect of perceived cuteness on intention to purchase through serial mediation (social presence, and user engagement) is drastically reduced. Thus, H6 was supported.

## Discussion

The cuteness of the AI voice assistant’s appearance plays an important role in the intention to purchase (Lu et al., 2021). Cuteness is a good perceptional concept that can strengthen the engagement between consumers and products and raise their engagement, making consumers more eager to purchase products. Based on social presence theory and user engagement theory, this study explores the different impact of AI voice assistant appearances on consumers’ perception of cuteness. Then, this study verified the influence of cuteness on customers’ intention to purchase and the serial-mediation effect of social presence and user engagement. Finally, the moderating effect of perceived risk is of service failure was discussed. The research verified the impact of cuteness on customers’ purchase intention and the serial-mediation role of social presence and user engagement. Finally, the moderating effect of perceived service failure risk is discussed. This study found that consumers perceived animal-like AI voice assistants to be more cute than round-eyed and normal-looking AI voice assistants. The findings are in line with previous studies that juvenile eyes or animal shapes trigger consumer’s perception of cuteness, then stimulating intention to purchase (Borgi and Cirulli, 2016).

Second, the results also showed that the cute appearance of AI voice assistants positively affects users’ purchase intention. Moreover, there is a mediating effect of social presence and user engagement in serial. When users perceive the cuteness of the AI voice assistant’s appearance, they will feel warmth and compassion and want to take care of it, which will promote their willingness to interact with the AI voice assistant and make them more likely to perceive the social presence of AI voice assistants, such as interact with a cute child or a kind friend. It is consistent with the previous findings (Sherman and Haidt, 2011). Users who treat an AI voice assistant as a social entity instead of a device will promote their engagement. Their cognitive, emotional, and behavioral aspects will be influenced (e.g., want to play a game with an AI voice assistant when they are bored, be cared for by an AI voice assistant when they are depressed, ask questions to an AI voice assistant when they are confused). AI devices are trated as a family member (Caudwell and Lacey, 2020) so they will accept AI voice assistant more easily in their daily life. The relationship between AI voice assistants and the user will become more intimate, and the user will want to have AI voice assistants more. It means that companies can consider actively designing cute product looks to stimulate consumers’ perception of the product and thus encourage them to interact with it actively, increasing their intention to purchase later.

Third, this study examined the moderating effect of perceived risk on the serial mediation model. The result showed that higher perceived risk could weaken the influence of perceived cuteness on intention to purchase through social presence and user engagement. AI is a new, developing, and advanced technology. The adoption of new technologies is slowed by the fact that the average person may not fully comprehend how the technology operates (Hussain et al., 2021). Higher perceived risk is often caused by time, money, and emotional loss due to the failure of AI voice assistant services. Once consumers have discovered that AI voice assistants could generate undesirable repercussions, they will avoid them by stopping to contact with AI voice assistants. Cuteness is a double-edged sword. Cute image will make people feel warm (Lu et al., 2021), feelings of healing (Chou et al., 2022), readiness to care, and social engagement (Borgi and Cirulli, 2016). On the other hand, people may perceive something cute as merely playful, whimsical, less serious, and lacking in gravitas or competence. According to McLean and Osei-Frimpong (2019), functional and social benefits, rather than hedonic ones, impact the use of AI voice assistants. Therefore, when the perceived risk is high, perceived cuteness of AI voice assistant may make users feel that AI voice assistant is less capable and is more likely to fail service.

## Contribution

### Theoretical implications

Firstly, this study advances the present literature by testing and supporting a moderated serial-mediation framework that social presence and user engagement sequentially mediate the association between perceived cuteness and intention to purchase. For the first time, this study examines the effect of social presence and user engagement on serial mediation. Previous studies only identified the influence of perceived cuteness on intention to purchase. Cuteness has been shown to be a significant indicator of consumer goodwill and brand loyalty (Lu et al., 2021), attitude toward the brand (Shen, 2020), and intention to purchase (Chou et al., 2022). Moreover, social presence plays a useful role in AI voice assistant adoption. Few studies have examined the cuteness effect in the AI voice assistant context. Leveraging the lens of social presence theory, our study filled in this research gap and clarified how cuteness affects a user ‘s intention to purchase in the AI voice assistant context.

Secondly, as there have been few prior research on the moderating influence of perceived performance risk, our study advances knowledge of the perceived performance risk mechanism in the pre-decision phase of AI voice assistant adoption. Despite perceived performance risk have been shown to have a significant role in the context of high technology products, to our knowledge, no research has yet examined the impact of perceived performance risk when it comes to AI voice assistants. Thus, this empirical study adds to the existing body of work on the adoption of high-tech products by investigating the moderating effects of perceived risk.

### Managerial contribution

This study helps AI voice assistant marketers and developers decide how to choose the appearance of AI voice assistants. The findings indicate that the appearance of cuteness can influence users’ intention to purchase through social presence and engagement. Marketers should consider the cuteness of an AI voice assistant’s appearance when developing a new product, as this will improve user interaction and a sense of closeness and emotional bonding, leading to good user engagement and intention to purchase.

Moreover, the marketers should more focus on the important role of users’ perception of performance risk to their products. The findings emphasize the moderating effects of perceived risk in the link between cuteness and intention to purchase. Higher levels of perceived risk may result in a weaker intention to purchase even the same product. When users have a high degree of perceived performance risk of AI voice assistant, high cuteness product may cause more service failure and reduce users’ desire to interact with AI voice assistant, whereas it will destroy user engagement and intention to purchase. The finding contributes to extant research that AI voice assistant is not the cutest, the better. The product design needs to grasp the degree of cuteness. At the same time, advertisements should try to highlight the superiority of product performance and reduce the risk of perception.

## Conclusion

This paper examines the following topics: (1) We investigate perceived cuteness’s direct positive effect on purchase intention. It can assist businesses in optimizing the appearance of adorable designs and adjusting their marketing impact. (2) We investigate the sequential mediation effect of social presence and user engagement between perceived cuteness and intention to purchase. The cuter the product’s design, the more consumers view their presence as that of a friend, encouraging engagement and boosting purchase intent. (3) We investigate the moderating influence of perceived risk. When the perceived risk is high, high cuteness products may be considered lacking ability and influenced interactive engagement and intention to purchase. It also reminds enterprises to grasp the product’s cuteness and avoid questioning ability and performance.

## Limitations and future research

Firstly, this study found that cuteness and the social presence of AI voice assistants have distinct societal benefits. Future research could further analyze the specific factors of AI-powered voice assistants, such as the perceived coolness of AI voice assistants, and examine the temperament of the assistant during the adoption and use of the technology. Secondly, this survey was confined to the consumers in China. This study’s results may alter if the model is retested in different situations or cultures. We need to do more research in the future to see if our findings hold up under scrutiny in a variety of settings and cultures. Finally, this study was undertaken in the setting of AI voice assistants. Future study may examine if our findings can be applied to other items, taking into account the impact of different product kinds and the varied features that people value (such as entertainment attributes or function attributes).

## Data availability statement

The raw data supporting the conclusions of this article will be made available by the authors, without undue reservation.

## Ethics statement

Ethical review and approval was not required for the study on human participants in accordance with the local legislation and institutional requirements. Written informed consent from the [patients/participants or patients/participants legal guardian/next of kin] was not required to participate in this study in accordance with the national legislation and the institutional requirements.

## Author contributions

XY contributed to the current research ideas and performed the statistical analysis. YS and XY wrote the first draft of the manuscript. ZX contributed to improving the manuscript. XY and XL edited the revised manuscript and contributed to avoiding language errors. All the authors contributed to the article and approved the submitted version.

## Funding

This research was supported by the Doctoral Research Foundation (XB2022018) from Liaoning University of Technology.

## Conflict of interest

The authors declare that the research was conducted in the absence of any commercial or financial relationships that could be construed as a potential conflict of interest.

## Publisher’s note

All claims expressed in this article are solely those of the authors and do not necessarily represent those of their affiliated organizations, or those of the publisher, the editors and the reviewers. Any product that may be evaluated in this article, or claim that may be made by its manufacturer, is not guaranteed or endorsed by the publisher.
